# Effects of Hemodynamic Differences on the Assessment of Inter-Brain Synchrony Between Adults and Infants

**DOI:** 10.3389/fpsyg.2022.873796

**Published:** 2022-06-03

**Authors:** Satoshi Morimoto, Yasuyo Minagawa

**Affiliations:** ^1^Keio University Global Research Institute, Keio University, Tokyo, Japan; ^2^Faculty of Letters, Keio University, Tokyo, Japan

**Keywords:** functional near-infrared spectroscopy, prewhitening, hyperscanning, wavelet coherence, infant, hemodynamic response function, synchrony

## Abstract

The simultaneous recording of brain activity in two or more people, termed hyperscanning, is an emerging field of research investigating the neural basis of social interaction. Hyperscanning studies of adult–infant dyads (e.g., parent and infant) have great potential to provide insights into how social functions develop. In particular, taking advantage of functional near-infrared spectroscopy (fNIRS) for its spatial resolution and invulnerability to motion artifacts, adult–infant fNIRS may play a major role in this field. However, there remains a problem in analyzing hyperscanning data between adult and young populations. Namely, there are intrinsic differences in hemodynamic time latencies depending on age, and the peak latency of the hemodynamic response function (HRF) is longer in younger populations. Despite this fact, the effects of such differences on quantified synchrony have not yet been examined. Consequently, the present study investigated the influence of intrinsic hemodynamic differences on wavelet coherence for assessing brain synchrony, and further examined the statistical removal of these effects through simulation experiments. First, we assumed a social signal model, where one counterpart of the dyad (e.g., infant) sends a social signal to the other (e.g., parent), which eventually results in simultaneous brain activation. Based on this model, simulated fNIRS activation sequences were synthesized by convolving boxcar event sequences with HRFs. We set two conditions for the event: synchronized and asynchronized event conditions. We also modeled the HRFs of adults and infants by referring to previous studies. After preprocessing with additional statistical processing, we calculated the wavelet coherence for each synthesized fNIRS activation sequence pair. The simulation results showed that the wavelet coherence in the synchronized event condition was attenuated for the combination of different HRFs. We also confirmed that prewhitening via an autoregressive filter could recover the attenuation of wavelet coherence in the 0.03–0.1 Hz frequency band, which was regarded as being associated with synchronous neural activity. Our results showed that variability in hemodynamics affected the analysis of inter-brain synchrony, and that the application of prewhitening is critical for such evaluations between adult and young populations.

## Introduction

Hyperscanning—the simultaneous measurement of brain activity from multiple individuals—is an efficient approach to assess the neural basis of social interaction. Since the first hyperscanning study in 2011 (Funane et al., 2011), many studies have used functional near-infrared spectroscopy (fNIRS) for hyperscanning. fNIRS is suitable for conducting hyperscanning experiments on social interaction because fNIRS Signals have sufficient spatial resolution on the cerebral surface and are less affected by electrical and motion artifacts than electroencephalography. Furthermore, fNIRS can be measured in more natural unconstrained settings than functional magnetic resonance imaging (fMRI) and magnetoencephalography (Balardin et al., 2017). These advantages of fNIRS enable us to conduct brain measurements during spontaneous social interactions, including eye contact, facial expressions, and gestures, in a realistic environment (Czeszumski et al., 2020). In particular, fNIRS is suited for measuring neural activity in young children (Lloyd-Fox et al., 2010) and is the best tool for conducting hyperscanning studies with infants (Minagawa et al., 2018).

Hyperscanning between parents and children is an important method for understanding how children acquire social skills through neural development and the parent–child relationship. Recently, researchers have begun to investigate inter-brain synchrony between parents and infants and between parents and young children. They reported that inter-brain synchrony increases during cooperative conditions (Reindl et al., 2018; Miller et al., 2019; Nguyen et al., 2021a), during holding infant (Minagawa et al., 2018), during free play with mutual gaze (Piazza et al., 2020), when engaging in verbal turn-taking conversations (Nguyen et al., 2021b), and during joint-watching of movie stimuli (Azhari et al., 2021). These studies support the view that neural synchrony between parents and children may play a key role in the development of social ability.

For fNIRS hyperscanning, wavelet coherence (Grinsted et al., 2004) has been one of the major analytical methods for assessing inter-brain synchrony since its introduction in an early study (Cui et al., 2012). Wavelet coherence quantifies the amount of synchrony between two signals in the time–frequency domain. Coherence ranges from 0 to 1, where 0 indicates no correlation and 1 indicates a perfect correlation between the signals at the corresponding time–frequency plane. Because the fNIRS signal (i.e., the hemodynamic response) is a non-stationary signal, wavelet coherence analysis is better suited for quantifying inter-brain synchrony than cross-spectrum analysis of the Fourier transform, which requires stationary signals (Issartel et al., 2006). The coherence values are typically averaged over the entire experimental block within a particular frequency band. It should be noted that wavelet coherence analysis in the hyperscanning literature implicitly assumes the same characteristic as in the dynamics of the paired signals; namely, wavelet coherence cannot correctly quantify the synchrony of paired activation sequences that have different frequency characteristics.

The hemodynamic response associated with neural activity is known to change along with brain maturation (Poppe et al., 2021). An fMRI study showed that an infant’s hemodynamic response function (HRF) has a small peak amplitude, long latency-to-peak, and a large undershoot (Arichi et al., 2012). For fNIRS, a delay in the peak latency (Minagawa-Kawai et al., 2011) and its decrease through infancy (Lloyd-Fox et al., 2017) have been reported. Furthermore, in addition to the commonly known canonical shape, inverted shapes (i.e., oxyhemoglobin concentration [oxy-Hb] showing a decrease and deoxyhemoglobin concentration [deoxy-Hb] showing an increase in their initial response) were often observed (Zimmermann et al., 2012; Issard and Gervain, 2018; Arimitsu et al., 2018). The reason for the variability in the HRF seems to be multifactorial (Issard and Gervain, 2018).

The hemodynamic activation pattern in the time–frequency domain depends on the shape of the HRF. Considering the fact that an infant’s HRF is different from that of an adult, wavelet coherence would underestimate the inter-brain synchrony between infants and adults. In the case of time-constrained social interaction tasks, even if the shapes of HRFs were different, synchrony could be detected in the band(s) of the task frequency (e.g., Miller et al., 2019). However, in the case of spontaneous social interactions, synchrony appears only in the bands corresponding to the frequency characteristics of the HRF. Moreover, the shape of an infant’s HRF is not well-characterized, so the estimation of the degree of reduction in coherence is difficult. It is necessary to develop a robust synchrony quantification method to take into account the variability in HRFs.

The aim of this study was to reveal the influence of hemodynamic differences between two signals on wavelet coherence. We conducted simulation experiments and investigated the extent to which hemodynamic differences distorted coherence. In particular, spontaneous social interactions between an adult and an infant were assumed. We set two conditions to assess the effect of neural synchronization on coherence: a synchronized-pair condition (i.e., neural activation patterns in the social brain regions of both participants were synchronized) and an unsynchronized-pair condition (i.e., there was no synchronization between a pair of neural activation patterns). We prepared several types of infant HRFs based on previous studies (see Materials and Methods). Simulated hemodynamic activity was synthesized for each HRF, and the wavelet coherence between the combination of adult and infant HRFs was calculated. Since there was a large difference in HRFs between the adult and infant, it was expected that several frequency bands of wavelet coherence would decrease even when the original neural activity patterns were highly correlated.

Furthermore, the present study aimed to seek a statistical method to remove the effects of hemodynamic differences. We focused on prewhitening to eliminate the effects of the HRF. Prewhitening is a valuable method for removing temporal autocorrelations of hemodynamic signals for the use of GLM analysis of fMRI (Friston et al., 2000; Woolrich et al., 2001). For fNIRS, prewhitening is useful for removing type I errors caused by the temporal autocorrelation of hemodynamic signals (Barker et al., 2013). Recently, Santosa et al. showed that prewhitening is important for the calculation of functional connectivity to reduce type I errors (Santosa et al., 2017). They also indicated that prewhitening was not only essential for correlation but also for wavelet coherence. We hypothesized that, if the order of whitening (i.e., how many previous time samples would be input to an autoregressive [AR] model) was set to a sufficiently large number, it would be possible to remove the serial correlation caused by both HRFs.

It should be noted that none of the fNIRS hyperscanning studies between adults and infants/children have taken the intrinsic hemodynamic differences between these individuals into account, despite the fact that differences in HRF potentially affect the results of wavelet coherence as stated above. To examine such effects, we chose a simulation-based approach, rather than an experimental approach. This is because it is not feasible to obtain an infant’s HRF with sufficient signal quality from an experiment which is completely free from artifacts (e.g., motion artifacts) so as to reasonably evaluate intrinsic HRF differences. This study aims to assess effects that are purely due to hemodynamic differences, within a realistic range. We, therefore, used synthetic hemodynamic activities based on the HRFs which had been reported in a previous experiment (see section Materials and Methods).

## Materials and Methods

### Social Signal Model

To assess the influence of HRF differences within dyads on wavelet coherence and the effect of prewhitening, we set several simulation experiments based on a social signal model. The social signal model was inspired by a previous study (Zhang et al., 2020). Zhang et al. explored an optimal computational approach to examine wavelet coherence of fNIRS signals. They conducted a simulation to determine the ideal coherence between two fNIRS signals with corresponding event sequences. We adopted their simulation approach and modified the simulation settings to fit our research purpose.

**FIGURE 1:**
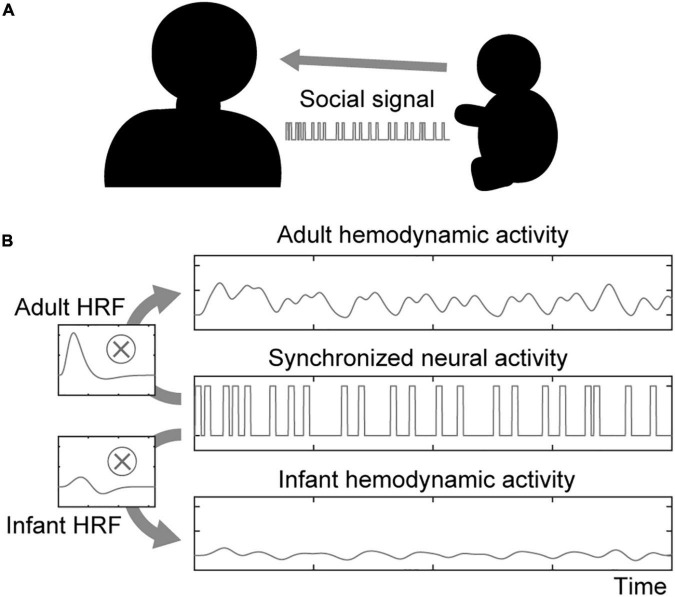
Illustration of the social signal model. **(A)** An interacting dyad comprising an adult and an infant is assumed in the social signal model. They send and receive social signals each other. **(B)** Neural activity in both the sender’s and receiver’s brains associated with social signal processing is synchronized upon sharing social signal events. The hemodynamic activity of each participant is modeled by boxcar-shaped neural activation convolved with the HRF.

In the social signal model, we considered a situation in which two participants of a dyad had a lively interaction with each other within a certain duration (Figure 1A). When one sends a social signal (e.g., eye contact, facial expression) to the other, the sender’s brain region related to social signal processing will be activated. Furthermore, if the receiver accepts the social signal from the sender, the receiver’s brain region related to social signal processing will also be activated. Hemodynamic activity is modeled by convolving neural activity with the corresponding hemodynamic function (Figure 1B). Note that the model can simulate the neural activations associated with social signals regardless of what the social signals are.

We assumed that the social signals were random and brief events similar to the perceptual detection of social cues (Zhang et al., 2020). Thus, the social signal event at time *t, s*(*t*), was designed as a boxcar function. The corresponding neural activity *n*(*t*) can be expressed as follows:


n(t)=s(t)+ε

where ε is the noise term. Similar to the design matrix in GLM analysis, hemodynamic activation *h*(*t*) can be modeled by convolving the neural activity with the HRF:


h(t)=n(t)×hrf

By exchanging the shape of the HRF, it is possible to obtain the corresponding hemodynamic activation.

### Hemodynamic Response Function

#### Double Gamma Model of the Canonical Hemodynamic Response Function

The most commonly used HRF is the canonical HRF, which is implemented in the SPM toolbox (Friston et al., 1998; Friston et al., 2007). The canonical HRF consists of a linear combination of two gamma functions to characterize the main response and undershoot, respectively:


$$f\left(t\right)=\frac{t^{\alpha_{1}-1}\beta_{1}^{\alpha_{1}}e^{-\beta_{1}t}}{\Gamma \left(\alpha_{1}\right)}-\frac{t^{\alpha_{2}-1}\beta_{1}^{\alpha_{2}}e^{-\beta_{2}t}}{c\Gamma \left(\alpha_{2}\right)}$$

where α_1_ is the delay of the response, α_2_ is the delay of the undershoot, β_1_ is the dispersion of the response, β_2_ is the dispersion of the undershoot, and *c* is the ratio of the response to the undershoot. The most popular setting of these parameters is the default setting of the SPM toolbox; α_1_ is set to 6 s, α_2_ is set to 16 s, β_1_ and β_2_ are set to 1, and *c* is set to 6.

#### Infant Hemodynamic Response Function

**FIGURE 2:**
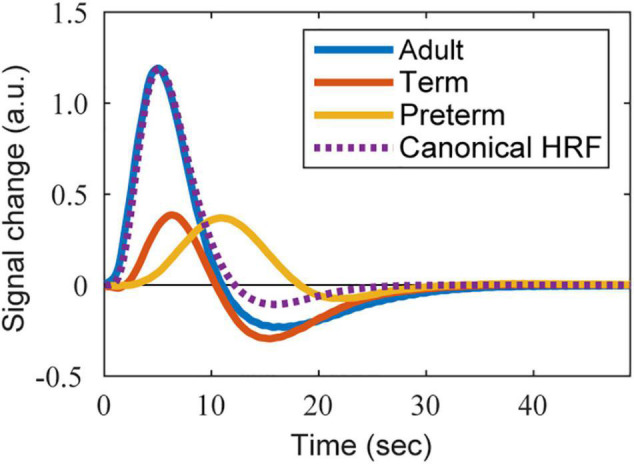
The shape of HRFs used in the simulation. The BOLD-based HRFs estimated from the BOLD responses reported in Arichi et al. (2012) are presented. Adapted from Arichi et al. (2012), with permission from Elsevier. Blue, red, and orange lines correspond to the HRFs of an adult, term infant, and preterm infant, respectively. The purple dotted line is the reference shape of the adult’s canonical HRF, whose height is adjusted to that of the estimated adult’s BOLD-based HRF.

To obtain the infant HRF, we utilized the BOLD response in the contralateral primary somatosensory cortex to 1 s of somatosensory stimulation (passive movement of the participant’s right hand), as described in a previous fMRI study (Figure 2 in Arichi et al., 2012). Two types of HRFs were used: one was obtained from 15 infants at term-equivalent post-menstrual age (PMA) (median 41±1 weeks) and the other was obtained from 10 preterm infants (median 34±4 weeks PMA). For further details, see Arichi et al., 2012. Twelve of the 15 infants at term-equivalent PMA had been born prematurely. Because many neuroimaging studies reported that both term and preterm neonates excluding very preterm neonates exhibit similar PMA-dependent development of brain connectivity and similar brain functions at term-equivalent age (Doria et al., 2010; Cao et al., 2017; Arimitsu et al., 2022), we included all 15 infants in the “term” group. The description of PMA-dependent development of the HRF pattern by Arimitsu et al. (2018) also supports this view. We carefully extracted the BOLD response curves of the term and preterm infants from the figure using WebPlotDigitizer (version 4.4; Rohatgi, 2021), and then resampled them at 10 Hz using MATLAB’s *resample* function with the linear interpolation setting. The data were low-pass filtered at 1 Hz to remove the high-frequency noise caused by the resampling process. The shapes of infant HRFs were fitted using a semiparametric smooth finite impulse response (FIR) model (Goutte et al., 2000) as performed in a previous study (Lindquist and Wager, 2007). For the FIR model fitting, we used the MATLAB script *Fit_sFIR*.m of *HRF_Est_Toolbox2*, which is available in the author’s GitHub repository.^1^ The fitted shapes of the HRFs of term and preterm infants are shown in Figure 2 (red and orange lines, respectively).

For validation purposes, we also investigated the influence of an inverted response on the wavelet coherence. We chose an inverted oxy-Hb response of newborn infants introduced in a previous fNIRS study (Figure 3 in Issard and Gervain, 2018), since a canonical response (i.e., a significant increase in oxy-Hb and decrease in deoxy-Hb as compared to baseline) was also reported under the same conditions. Coherence is an absolute value and ignores the phase; inversion is therefore not expected to affect coherence. As opposed to the BOLD response data, the oxy-Hb response data were induced by longer stimulus durations (i.e., 18 s) and did not show a smooth curve, resulting in suboptimal FIR model fitting. To resolve this issue, we attempted to fit the curve with a parametric model; after resampling from the figure, the data were smoothed using a 50 time-point (i.e., 5 s) moving average filter and were then regressed using a single gamma model to obtain the main peak response term in the canonical HRF. The parameters were optimized for maximum correlation using a grid-search method. The parameter α_1_ ranged between 0 and 30 s with 1-s steps, and parameter β_1_ ranged between 1 and 10 with steps of 1 unit (Supplementary Figure 1). The fitted canonical and inverted HRFs are presented in Supplementary Figure 2 (red and orange lines, respectively).

**FIGURE 3:**
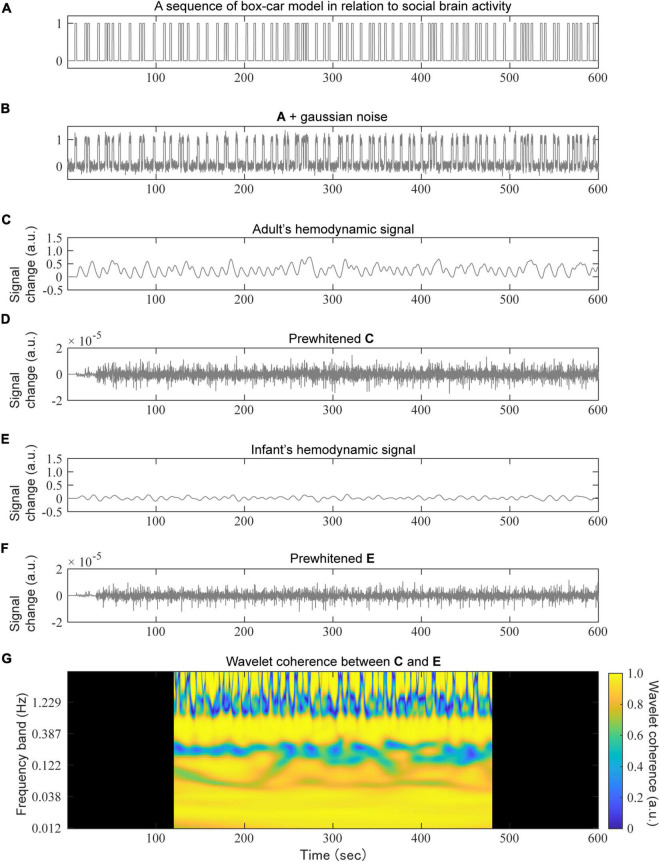
An example of a simulation based on the social signal model. A randomly generated social event sequence with a box-car model **(A)** to which a small Gaussian noise was added **(B)**, convolved with the adult’s HRF **(C)** or infant’s HRF **(E)** to obtain a hemodynamic signal sequence. For the prewhitening test, an AR (100) filter was applied to the hemodynamic signals of both the adult **(D)** and infant **(F)**. Finally, the wavelet coherence between **(D,F)** was calculated **(G)**. The dark areas in panel **(G)** were removed for the subsequent mean calculations.

The fitted BOLD-based and oxy-Hb-based HRFs are available in Supplementary Data 1, 2, respectively.

#### Adult Hemodynamic Response Function

We prepared two types of HRFs for adults: one corresponding to the BOLD responses and the other corresponding to the oxy-Hb responses. The BOLD-based HRF was estimated from the data acquired in the same study as that reporting the infant responses (Arichi et al., 2012) by fitting an FIR model as described above. The original BOLD response was obtained from 10 healthy adults (median age 31.5 years). The fitted HRF is shown in Figure 2 (blue line).

For the oxy-Hb-based HRF, we adopted the canonical HRF with the default settings of the SPM toolbox, but only used the term of the main response (i.e., a single gamma function). This was because an undershoot was not observed in the infant HRF of the OxyHb signals. The shape of the oxy-Hb-based HRF is shown in Supplementary Figure 2 (dotted blue line).

As with the infant HRFs, the fitted BOLD-based and oxy-Hb-based HRFs are available in Supplementary Data 1, 2, respectively.

### Simulation Experiments

We carried out simulation experiments to show the extent to which the wavelet coherence would be affected by differences in the HRFs (Figure 3). All simulations were performed using MATLAB R2021a (MathWorks Inc., MA, United States).

We considered two conditions for the neural activation sequence pair. The first is the synchronized-pair condition in which the same neural activation is used but the convoluted HRFs are different. The second is the unsynchronized-pair condition, in which the absolute value of the correlation coefficient between the neural activity patterns satisfies |*r*| < 0.01.

We generated 1,000 random neural activation sequences for each condition. The length of a neural activation sequence was set to a total of 12 min, with 1-min blank periods at both the beginning and end of the sequence (Figure 3A). The duration of each boxcar-shaped social signal event, with a height of 1, was 2 s. The number of events was set between 20 and 140, with steps of 20. Note that the setting corresponding to the previous study (Zhang et al., 2020) was 80 events. The noise in the neural activation sequence was modeled using white Gaussian noise with a variance 0.1 (Figure 3B).

Each neural activation sequence was convolved with several types of HRFs to obtain the corresponding hemodynamic activation sequence (Figures 3C,E). As mentioned above, we prepared two combinations of HRF types (namely, the BOLD-based HRF and the oxy-Hb-based HRF), and each HRF-type condition included both adult and multiple-infant HRFs. We calculated wavelet coherence between adult hemodynamic activation and the corresponding infant activation (Figure 3G), but also between another adult hemodynamic activation (i.e., with different Gaussian noise) as a control. For the calculation of wavelet coherence, we used the *wcoherence* function in MATLAB with default settings: there were 12 voices per octave. The total number of frequency bands was 100. The upper and lower limits of the frequency bands were 3.68 and 0.012 Hz, respectively. To remove the boundary effects of the outside of the cone of influence, 2-min periods both at the beginning and the end were removed. We calculated the average of the wavelet coherence through complex values along the time domain for each frequency band (Zhang et al., 2020).

Note that for all frequency bands, the optimal value of the wavelet coherence in the synchronized-pair condition and in the unsynchronized-pair condition was 1 and 0, respectively. However, the small white Gaussian noise eliminated coherence at a higher frequency band. Moreover, the coherence at a lower frequency band tended to show a larger value on average. Thus, the coherence of the unsynchronized-pair condition should be considered as a baseline value.

For prewhitening, an AR model-based filtering method was utilized (Barker et al., 2013). We used a customized version of the *ar_fit* function, which was distributed in the NIRS Brain AnalyzIR Toolbox (Santosa et al., 2018),^2^ and invalidated the estimation process of the order of the AR model using the Bayesian information criterion (BIC) to avoid the difference between the orders of paired data affected by the wavelet coherence. Prewhitening was applied before calculating the wavelet coherence (Figures 3D,F). The order of the AR model was set to 100 samples (i.e., 10 s) for all hemodynamic sequences in our simulations. This is because the interpretation of the coherence value would be complicated if the estimated orders were different between the paired hemodynamic sequences. To quantify the influence of prewhitening on wavelet coherence, the effect size was calculated for each dyad condition and each frequency band using Hedge’s *g* (Hedges, 1981).

## Results

### BOLD-Based Hemodynamic Response Functions

**FIGURE 4:**
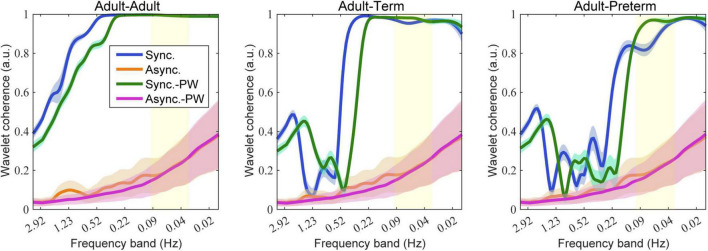
Simulation results of wavelet coherence with 80 social events. The colored lines and areas represent the mean and standard deviation of wavelet coherence, respectively. Blue indicates the synchronized-pair condition, orange indicates the asynchronized-pair conditions, magenta indicates the synchronized-pair condition with prewhitening, and green indicates the asynchronized-pair conditions with prewhitening. The yellow area indicates the targeted frequency bands generally used for wavelet coherence analysis (0.03–0.1 Hz).

Typical examples of the simulation results are shown in Figure 4. As illustrated, the averaged wavelet coherence decreased in the adult–infant dyads in the synchronized-pair condition. The dyad comprising an adult and a preterm infant tended to show smaller coherence than that with an adult and a term infant. Prewhitening before the calculation of wavelet coherence recovered the averaged wavelet coherence in frequency bands below 0.1 Hz, especially for the dyad comprising an adult and a preterm infant. For the dyad comprising adults, prewhitening did not clearly affect coherence in frequency bands below 0.1 Hz.

**FIGURE 5:**
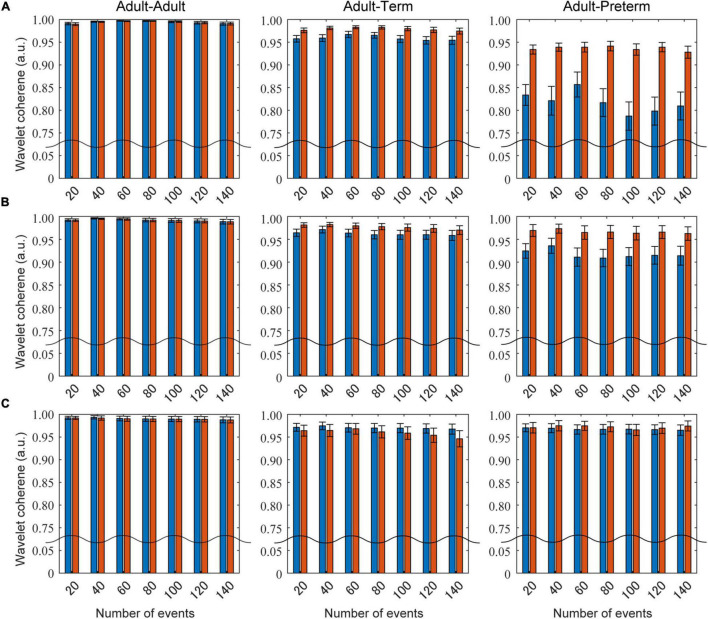
The influence of the number of social events on wavelet coherence. Blue bars show the mean coherence without prewhitening, and red bars indicate that with prewhitening. Panels **(A–C)** are the results at the 0.086, 0.048, and 0.030 Hz frequency bands, respectively.

Figure 5 presents the influence of the different settings of the number of social events on the wavelet coherence at the 0.086, 0.048, and 0.020 Hz frequency bands. Prewhitening recovered the coherence for certain values in the adult–term and adult–preterm dyad conditions at 0.086 and 0.048 Hz. There was no clear relationship between the number of social events and coherence.

**FIGURE 6:**
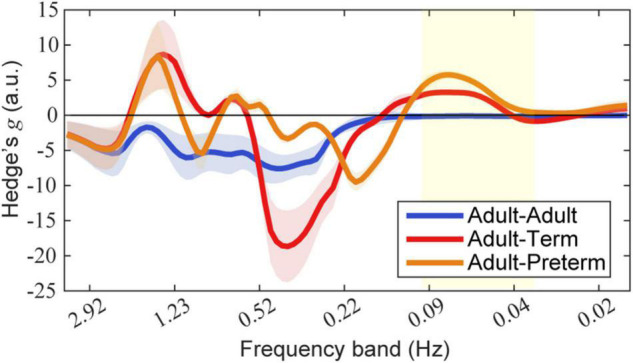
Effect size of prewhitening on wavelet coherence. Each mean and standard deviation of Hedge’s g over seven types of event-number conditions are presented as a line and shaded area, respectively. Blue, red, and orange lines indicate dyads of adult–adult, adult–term, and adult–preterm conditions, respectively. The yellow area indicates the targeted frequency bands (0.03–0.1 Hz). Note that a positive value indicates that prewhitening led to an increase in coherence.

The effect size of prewhitening for each dyad condition is shown in Figure 6. The results demonstrate that, for both the adult–term dyad and the adult–preterm dyad, prewhitening increased the coherence in the 0.03–0.1 Hz frequency band; this was mainly referred to as neurogenic activation in previous studies (Stefanovska, 2007; Zhang et al., 2020).

### Oxy-Hb-Based Hemodynamic Response Functions

Similar to results with the BOLD-based HRFs, the averaged wavelet coherence between the adult and infant showed relatively smaller values and was recovered by prewhitening in the frequency bands related to neural activation (Supplementary Figures 3–5). As expected, even when the shape of the HRF was inverted, prewhitening could recover coherence. However, in contrast to the result of the BOLD-based HRF, prewhitening clearly reduced the coherence between the adult–adult dyad for all frequency bands.

## Discussion

We addressed the influence of hemodynamic differences in fNIRS signals on wavelet coherence. We conducted simulation experiments based on the social signal model, which modeled spontaneous social interactions within a dyad. The strength of neural synchrony of adult–child dyads was assessed because there was a large difference in their typical HRFs. The results of wavelet coherence showed that a hemodynamic difference caused an inaccurate quantification of neural synchrony. To resolve this problem, we proposed the addition of prewhitening via an AR model-based filtering as a pre-processing step. As expected, further simulations indicated that prewhitening could recover the attenuation of coherence in the frequency bands corresponding to neurogenic activation.

Hemodynamic variability is a well-known issue in fMRI studies, not only for infant HRFs (Arichi et al., 2012) but also for the HRFs of adults (Aguirre et al., 1998). To allow the variations in the shape of the HRF for GLM analysis, the partial derivatives of the canonical HRF with respect to its peak delay and dispersion parameters were added as further basis functions (Friston et al., 1998). For fNIRS, the canonical HRF of the BOLD signal has been conventionally utilized in the adult hemodynamic response model (Ye et al., 2009). However, the shape of the HRF depends on the task type (Uga et al., 2014) and varies between different brain regions (Hong and Nugyen, 2014). In particular, the shape and latency of HRFs in young populations differed greatly, leading to the proposal of reconstructing the HRF of the infant before engaging in GLM modeling (Minagawa-Kawai et al., 2011). The mismatch of the shape of the HRF decreases the detection performance in the GLM analysis (Ciftçi et al., 2008). Similarly, we found that hemodynamic variability affects the amount of estimated inter-brain synchrony, particularly between an adult and an infant.

In our previous study (Minagawa et al., 2018), we briefly reported a preliminary result of mother–infant inter-brain synchrony when the mother held her infant. We used independent component analysis (ICA) to extract common independent components that were shared between hemodynamic signals. However, considering the large difference in hemodynamics between adults and infants, ICA was not optimal for evaluating the actual power of inter-brain synchrony. To date, we have examined several approaches to solve this issue; one possible method is the use of nonnegative matrix factorization (NMF) (Minagawa, 2017; Morimoto et al., 2018). NMF decomposes a non-negative matrix into two smaller non-negative matrices, a basis matrix and a coefficient matrix, whose product approximates the original matrix (Berry et al., 2007). NMF is a popular method in the acoustic research field (e.g., audio source separation) (Virtanen, 2007; Wang and Zhang, 2012). First, we calculated the power spectrograms of the hemodynamic signals and combined them into a large non-negative matrix. Then, the matrix was decomposed using NMF. If there was a common activity pattern between the channels and frequency bands, they were combined into a component. However, while the estimation of the optimal number of components is critical for NMF, it is practically unstable because fNIRS signals during interactions are usually noisy. Consequently, wavelet coherence would be more suited for analyzing data obtained from adult–infant or adult–child dyads.

Prewhitening is a classic method for removing serial correlations in the target signal; however, it has recently been used for fNIRS signal processing (Barker et al., 2013; Santosa et al., 2017). We hypothesized that if enough serial correlation was removed, the influence of the HRF difference would be canceled out. In our simulations, we utilized the AR model-based filtering method and manually set the order of the AR model to 100 (i.e., 10 s at 10 Hz). Santosa et al. found that using a BIC model selection, a model order of 10 to 20 was sufficient for resting state data recorded at around 5 Hz (Santosa et al., 2017). Meanwhile, Blanco et al. reported that the range of the optimal order varied between 60 and 110 at a sampling rate of 8.93 Hz (Blanco et al., 2018). Our selected order was similar to that used in the latter study. It should be noted that the optimal order depends on the properties of the target signal (e.g., signal-to-noise ratio). In real fNIRS datasets, motion artifacts and other sources of environmental noise are generally included. Moreover, the actual shape of the HRF evoked by a social signal is unknown. Our simulation results suggest that if the order of the AR model is sufficiently large, it is possible to recover the expected coherence.

We calculated wavelet coherence between dyads comprising adults as a control. Our simulation results of BOLD-based HRFs showed that prewhitening did not affect coherence in the frequency bands related to neural activation. However, in the oxy-Hb-based HRF, prewhitening slightly reduced the coherence across all frequency bands. This was probably caused by the synthetic shape of our HRF (we used a single gamma function); AR filtering is unsuitable for non-negative time series. Considering the regional differences in hemodynamics, even for hyperscanning data of adult dyads, prewhitening is recommended to remove the effect of the HRF as well as to reduce type 1 errors (Santosa et al., 2017).

For hyperscanning data analysis, there are several methods for quantifying social interactions (Czeszumski et al., 2020). Because each measurement system has specific characteristics, it is important to examine whether each method is valid for a specific purpose. The fNIRS signal quantifies the hemodynamic signal, and so the hemodynamic characteristics should be considered in the choice of analytical method and its development. Wavelet coherence is the most popular method; however, as our simulations indicated, it is sensitive to the hemodynamic characteristics of the fNIRS signal.

The HRF model for infants chiefly employed in the present simulation analysis was adapted from Arichi et al. (2012), which was based on somatosensory responses without much response delay in term infants. However, as mentioned before, the delay in infant HRFs differed greatly depending on the brain region, task, and infant sleeping state. Thus, a slower latency in infant HRFs, similar to the preterm case in this study, even for older infants, would be expected, as exemplified by the 2.8-s delay in the auditory responses of 4-month-old infants (Minagawa-Kawai et al., 2011). It can also be reasoned that the higher the task level in terms of cognitive aspect, the slower would be the brain response in infants, because higher brain function develops slowly. This means that even for older infants, the application of prewhitening to a dataset of social interactions is recommended.

In this study, we conducted simulation experiments, not real fNIRS measurements. To experimentally support our current findings, two fNIRS experiments are needed: the first to obtain adult and infant HRFs, and the second a random stimulation task, as performed by Zhang et al. (2020). However, performing such experiments with infants is quite difficult because fNIRS signals of sufficient quality are required to obtain a clear HRF. Although analysis methods have advanced considerably, fNIRS analysis, particularly for infants is still immature and one cannot remove all experimental artifacts. Because the simulation experiments were sufficient to demonstrate both the ideal decrease in coherence caused by intrinsic hemodynamic differences and the influence of prewhitening, we decided here to perform only the simulation experiments. We hope that the proposed methods can be verified with experimental fNIRS datasets in the future.

Finally, it should be emphasized that the simulation results strongly depend on the simulation settings. Our social signal model assumes that some social signals are shared between a dyad. This simple assumption is sufficient to explain the synchronization of the hemodynamic responses between the components of a dyad. As a consequence, however, the model cannot guarantee that the two components have interacted (e.g., when the two components respond to the same external signal). Note that synchronized behavior, even synchronized brain activity, is not evidence of the exchange of information between the members of a dyad (Hasson et al., 2012; Hasson and Frith, 2016). In the future, we expect to develop another model for the evaluation of bidirectional interactions, which can verify social cognitive concepts such as the we-mode (Gallotti and Frith, 2013).

## Data Availability Statement

The raw data supporting the conclusions of this article will be made available by the authors, without undue reservation.

## Author Contributions

SM: performed the simulation experiments. SM and YM: analyzed the data and wrote the manuscript. Both authors contributed to the article and approved the submitted version.

## Conflict of Interest

The authors declare that the research was conducted in the absence of any commercial or financial relationships that could be construed as a potential conflict of interest.

## Publisher’s Note

All claims expressed in this article are solely those of the authors and do not necessarily represent those of their affiliated organizations, or those of the publisher, the editors and the reviewers. Any product that may be evaluated in this article, or claim that may be made by its manufacturer, is not guaranteed or endorsed by the publisher.

## Footnotes

1
https://github.com/canlab/CanlabCore.git

2
http://huppertlab.net/nirs-toolbox-2/
