# Supplementary material for: Effects of Hemodynamic Differences on the Assessment of Inter-Brain Synchrony Between Adults and Infants
Source: Front Psychol. 2022 Jun 3;13:873796. doi: 10.3389/fpsyg.2022.873796 (PMC9205639; doi:10.3389/fpsyg.2022.873796)
Supplement: Supplementary file 3 [file Data_Sheet_3.docx]

Supplementary Material


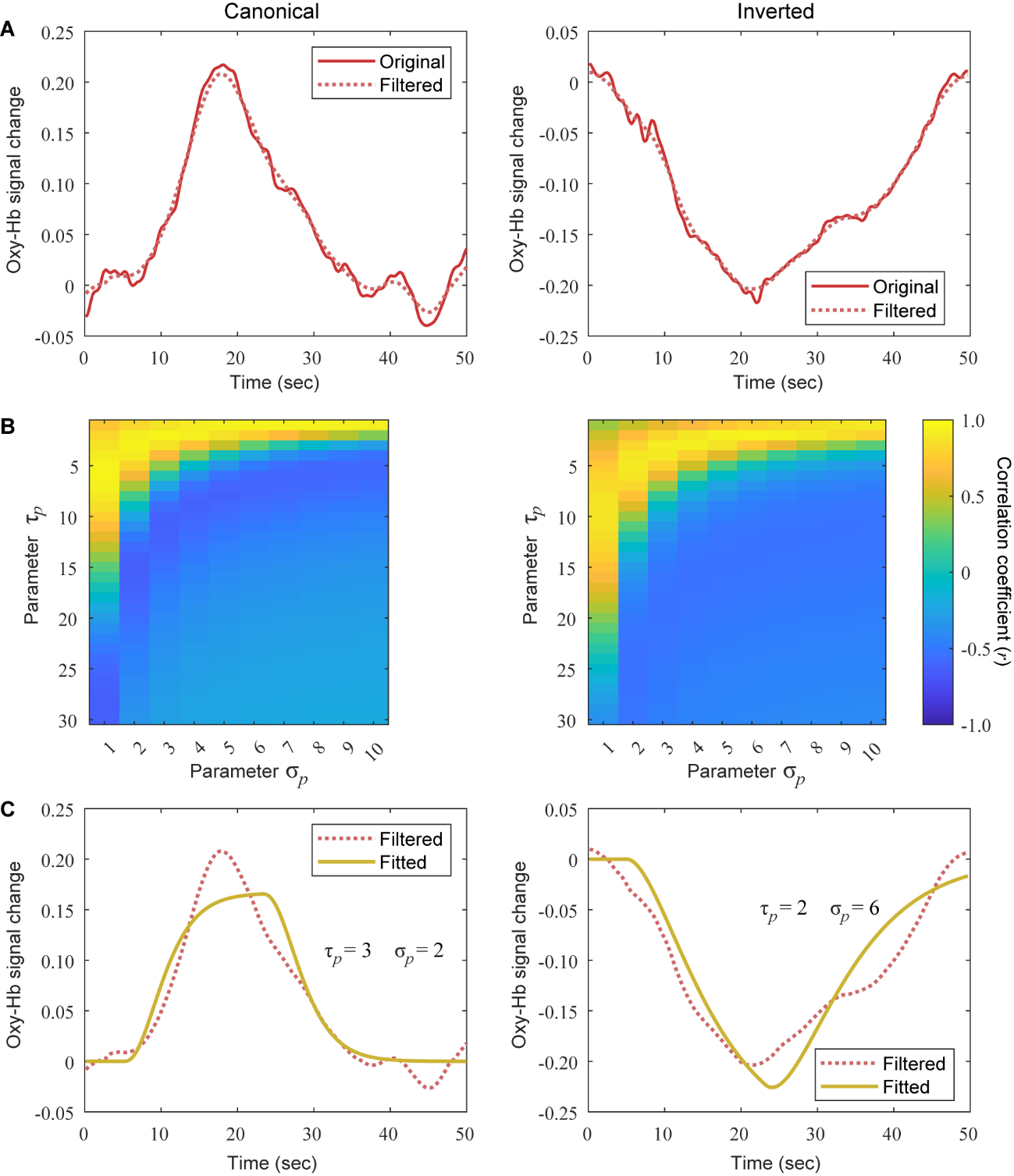


**Supplementary Figure 1.(A)** The red solid line and red dotted line indicate the original oxy-Hb signal response, which has been reported in Issard et al. (2018), and the filtered (i.e., moving averaged) response, respectively. The right panels indicate the canonical pattern, and the left panels indicate the inverted pattern. **(B)** Colored map of the correlation coefficients between the filtered response and the simulated responses obtained using the single gamma model. **(C)** The yellow solid line is the simulated response obtained by implementing the single gamma model with the most fitted parameter set (for the canonical pattern, $\tau_{p}=3$ and $\sigma_{p}=2$; for the inverted pattern, $\tau_{p}=2$ and $\sigma_{p}=6$). The red dotted line is the same as the one in **(A)**. Adapted from Issard et al. (2018), with permission from Elsevier.


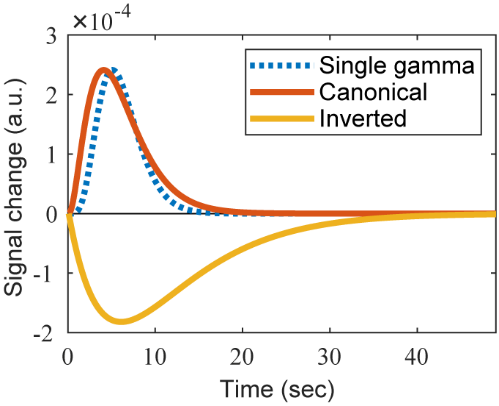


**Supplementary Figure 2.** The fitted oxy-Hb-based HRFs using the plot from Issard et al. (2018), with permission from Elsevier, are shown. The red and orange lines correspond to the canonical pattern and inverted pattern of the infant’s HRFs, respectively. The blue dotted line is the adult’s HRF modeled using the single gamma function, whose height is adjusted to that of the fitted infant’s canonical HRF.

**
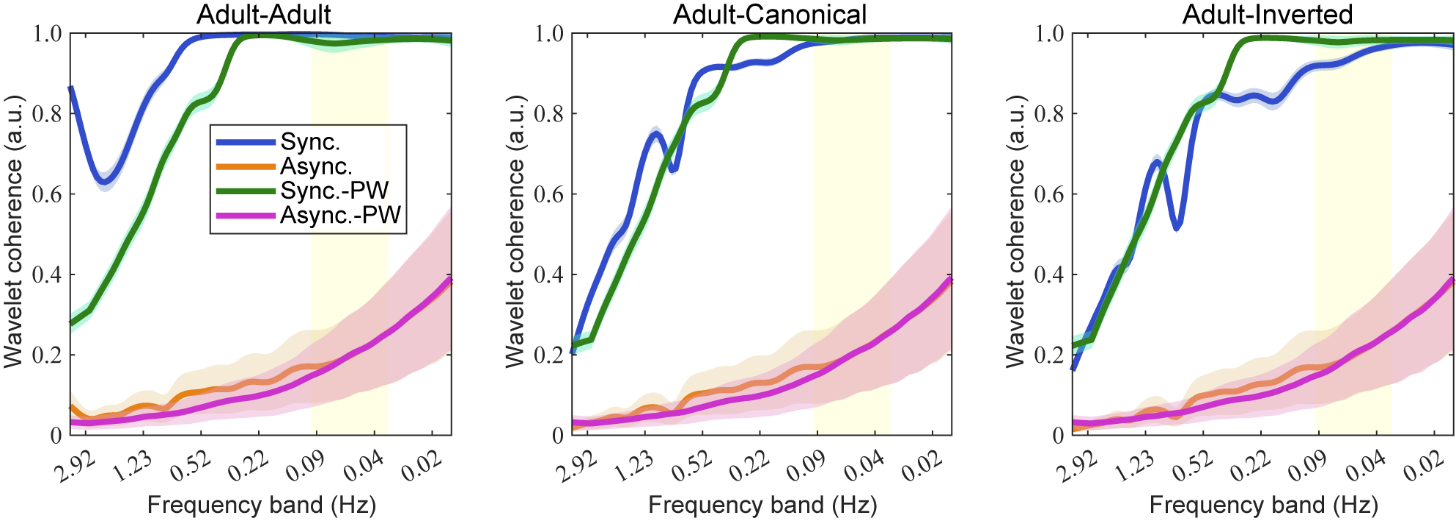
**

**Supplementary Figure 3.** Simulation results of wavelet coherence with the oxy-Hb–based HRFs and a social-event number of 80.Each colored line and area represent the mean and standard deviation of wavelet coherence, respectively. Blue indicates the synchronized-pair condition, orange indicates the asynchronized-pair conditions, magenta indicates the synchronized-pair condition with prewhitening, and green indicates the asynchronized-pair conditions with prewhitening. The yellow area indicates targeted frequency bands (0.03–0.1 Hz).

**
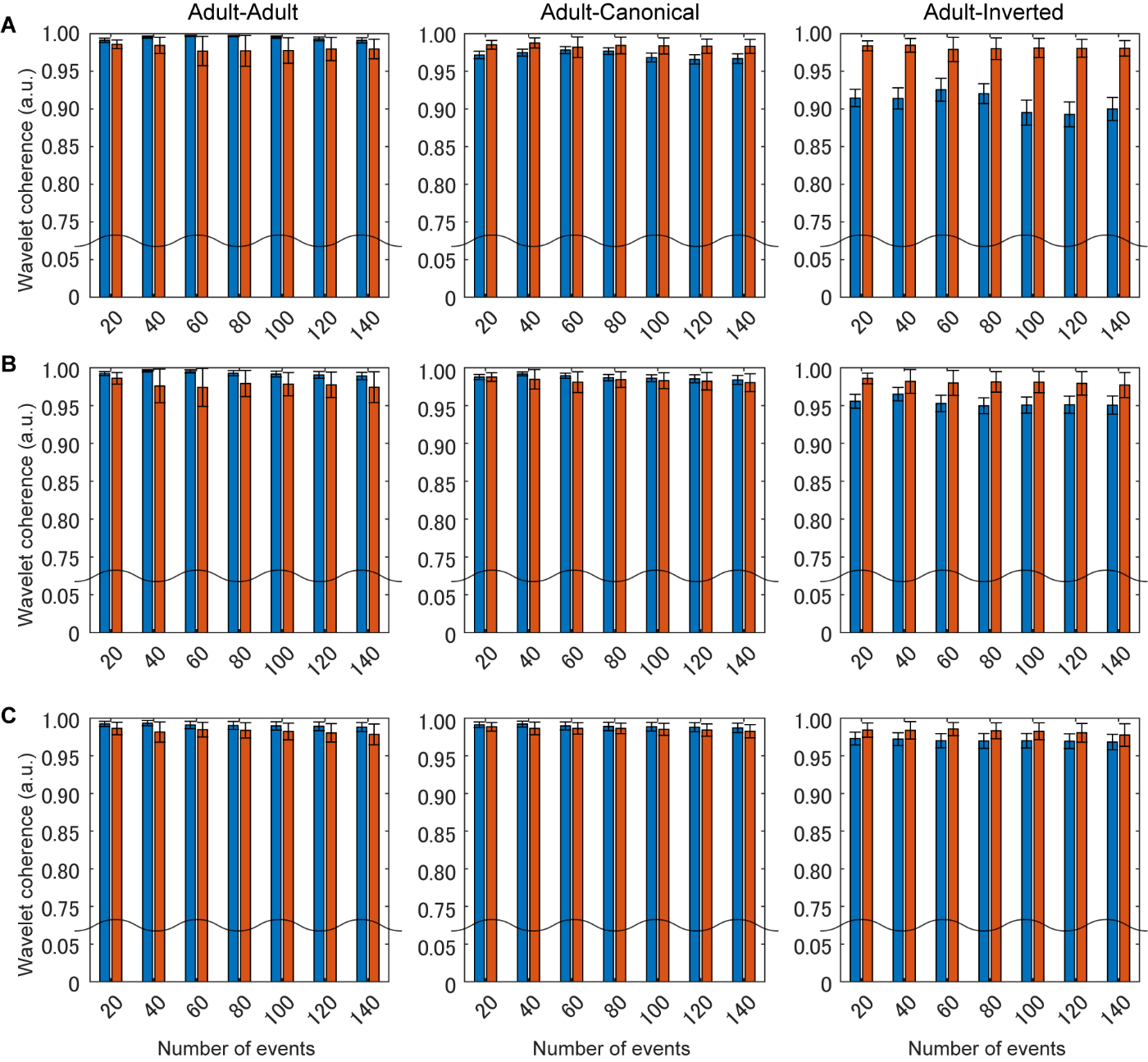
**

**Supplementary Figure 4.** The influence of the number of social signal events on wavelet coherence when using the oxy-Hb–based HRFs. Blue bars indicate the mean coherence without prewhitening, and red bars indicate that with prewhitening. **(A)**, **(B)**, and **(C)** present the results at the 0.086 Hz, 0.048 Hz, and 0.030 Hz frequency bands, respectively.

**
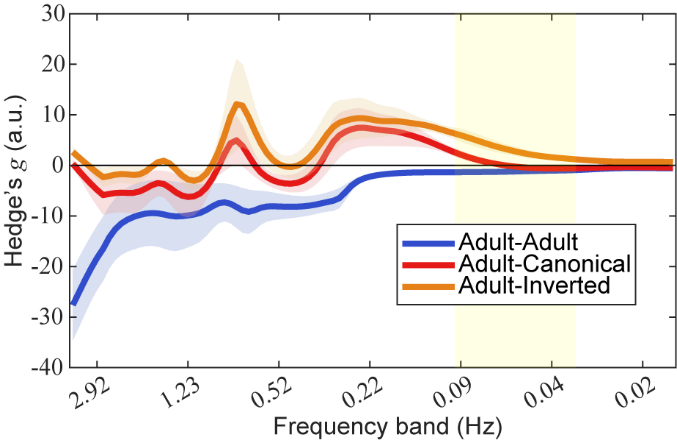
**

**Supplementary Figure 5.** Effect size of prewhitening on wavelet coherence when using the oxy-Hb–based HRFs. Blue, red, and orange lines indicate adult–adult, adult–canonical, and adult–inverted dyad patterns, respectively. The yellow area indicates targeted frequency bands (0.03–0.1 Hz).
